# PatchView: A Python Package for Patch-clamp Data Analysis and Visualization

**DOI:** 10.21105/joss.04706

**Published:** 2022-10-09

**Authors:** Ming Hu, Xiaolong Jiang

**Affiliations:** 1 Department of Neuroscience, Baylor College of Medicine, Houston, TX; 2 Jan and Dan Duncan Neurological Research Institute at Texas Children’s Hospital, Houston,TX, USA; 3 Department of Ophthalmology, Baylor College of Medicine, Houston, TX

## Summary

PatchView is a Python package for visualizing and analyzing multi channel patch-clamp (multi-patch) data (Neher & Sakmann, 1992), including intrinsic membrane properties and firing pattern analysis (Komendantov et al., 2019; McKinney et al., 2022), mini-event analysis(Clements & Bekkers, 1997), synaptic connection detection (Jiang et al., 2015), morphological analysis (Palacios et al., 2022) and more.

PatchView integrates multiple open-source tools and provides an intuitive graphic user interface (GUI) for navigating through the data files and analyses pipeline. It is aimed to enable users to perform most analysis quickly for the data collected in a typical patch-clamp experiment without managing a Python environment or writing any Python scripts.

Please refer to the full documentation and the source code for detailed information.

## Statement of Need

### Research purpose

PatchView was designed to be used by neuroscientists for handling electrophysiology data recorded from cells in alive tissues (such as brain slice or cultured cells) in patch-clamp experiments. The target audience is anyone working with patch-clamp data, with or without programming skills.

### Problems solved

**Figure 1:**
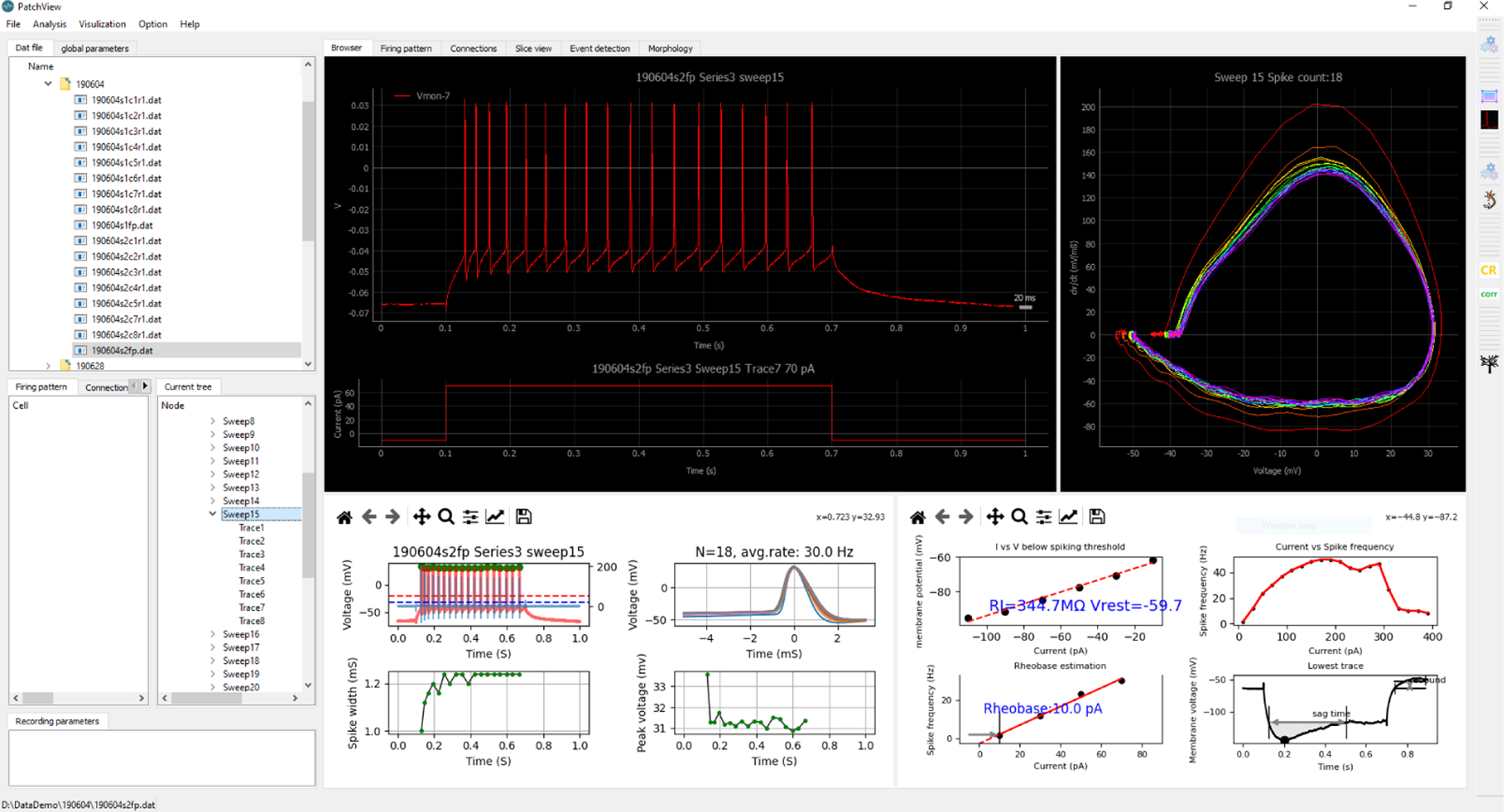
PatchView graphic user interface. Top left: file browsers. Middle left: experiments data inner trial selection. Right: multiple plots during a typical analysis). The toolbar is seen on the far right edge of the interface.

**Figure 2:**
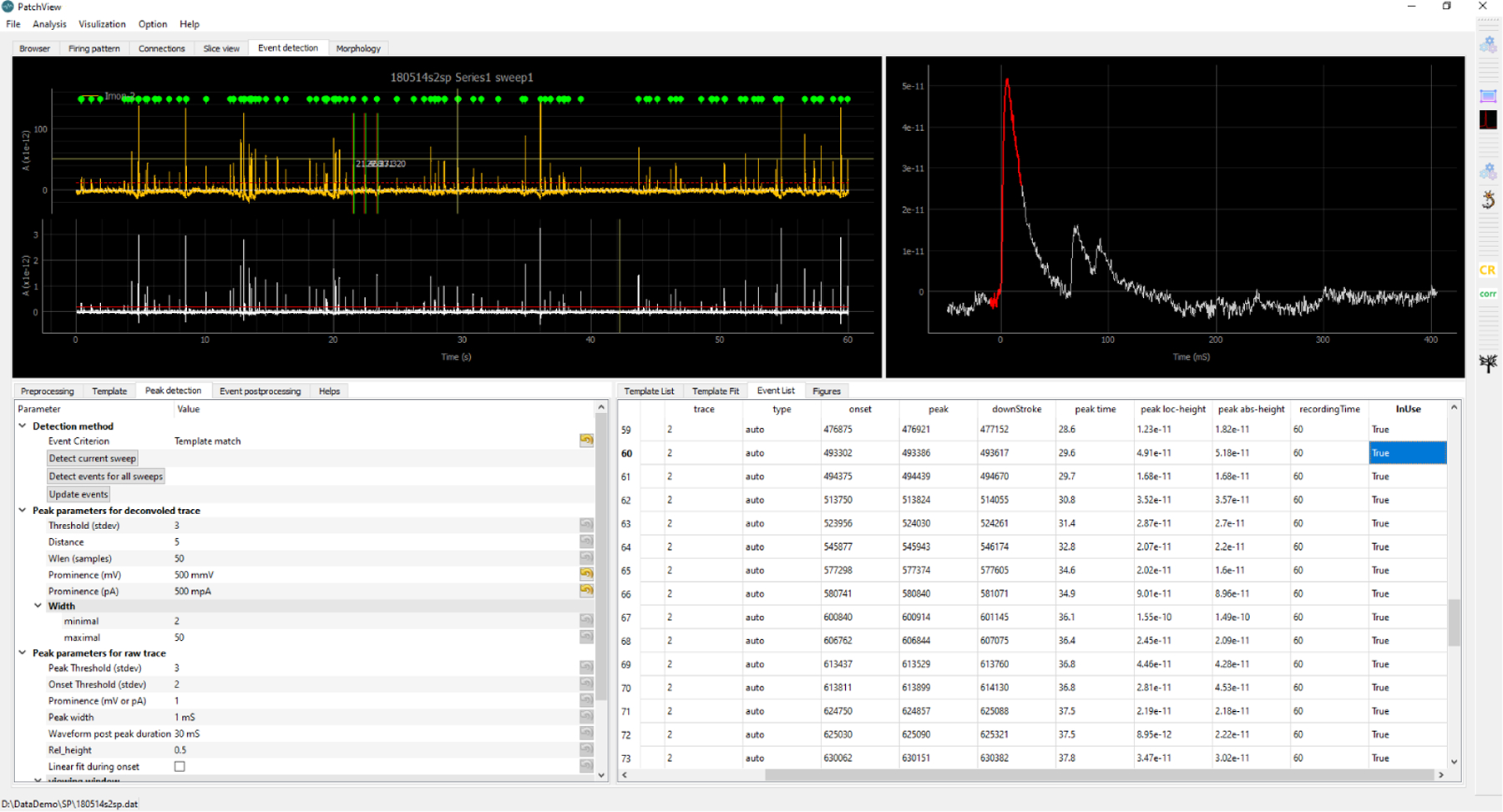
PatchView mini-event analysis GUI. Top left: large-scale view of the trace. The top yellow line is the original trace. The bottom white trace is a deconvolved trace with separate threshold (red horizontal line). Top right: currently selected event. Bottom left: parameters panel. Bottom right: table for detected events’ detailed quantification.

**Figure 3:**
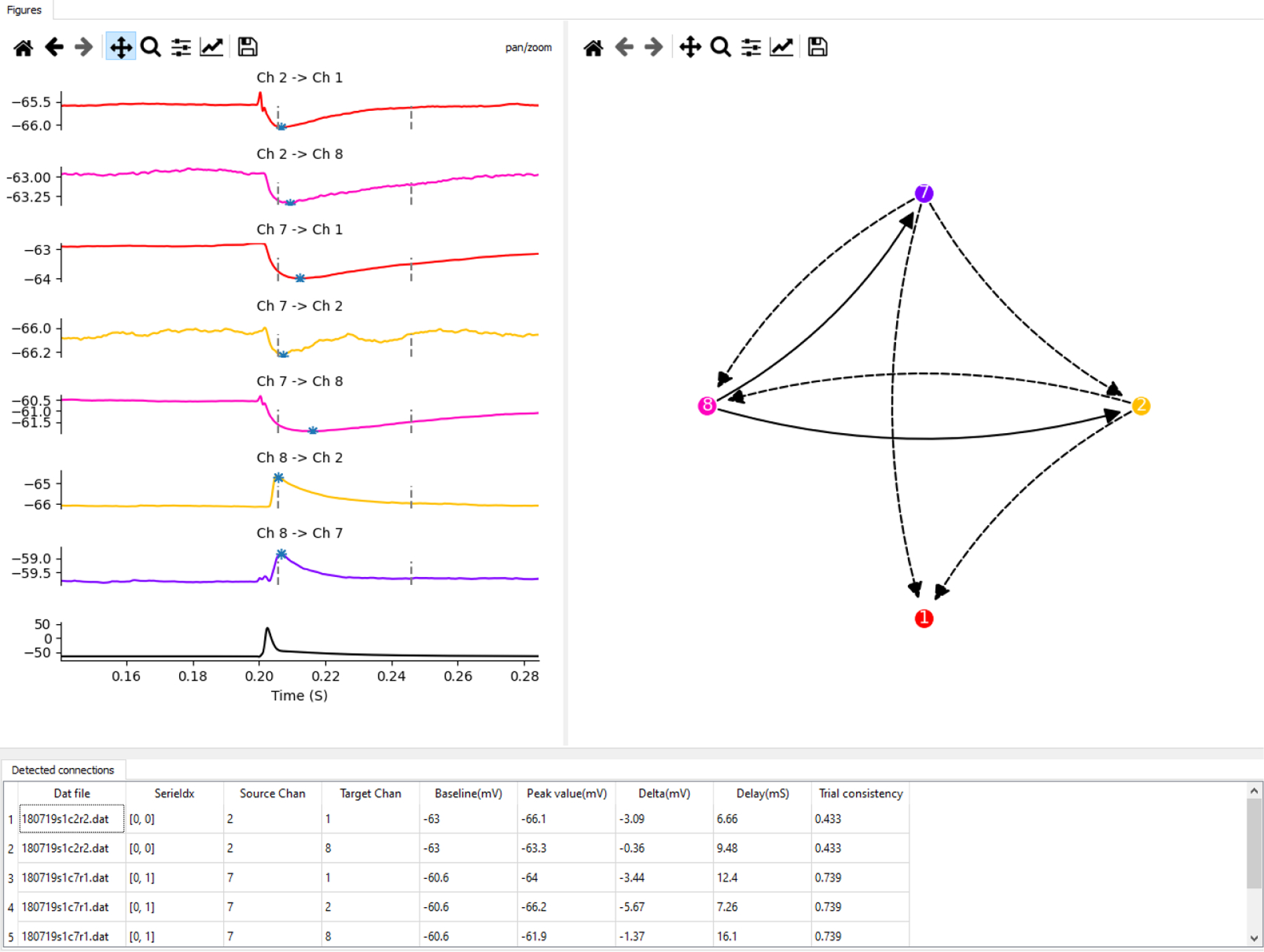
PatchView synaptic connection analysis GUI. Middle left: data series browser. It lists series recorded in a set of synaptic connection experiments from a group of neurons simultaneously recorded in the same slice. Top right: the left plot shows averaged traces of stimuli invoked responses. titles show the channel names of pre and post neurons. The right plot is a graph representation of detected connections. Bottom right panel: quantification of detected connections: including peak voltage, peak height, peak delay, trial consistency.

Main functionalities of PatchView:

- Importing both Heka data and Axon Instruments data. Exporting to Python pickle file or NWB (Neurodata Without Borders) file format (Rübel et al., 2022).
- Visualizing single and multiple traces with zoom, pan operations.
- Automatically sorting experiments data according to predefined labels.
- Performing analysis on intrinsic membrane properties, action potential detection, firing pattern analysis (Figure 1).
- Synaptic connection analysis (Figure 3).
- Mini-event analysis (Figure 2).
- Visualizing and quantification of neuron’s morphological reconstruction from Neurolucida

### Compares to other commonly-used packages

Commercial software such as Patchmaster, Clampfit provide rich functions for handling this type of data. However, the former only supports Heka data, while the latter only support Axon Instruments data. PatchView supports both .dat (from Heka) and .abf format (from Axon Instruments). To facilitate data sharing, PatchView could export imported data as NWB file.

Stimfit(Guzman et al., 2014) is a well-known python package for dealing with pre/post synaptic events in single channel. Compared to Stimfit, PatchView also provides more intuitive user interface (Figure 2) and more native support for Heka dat file. For instance, most of the time, a Heka dat file may host data recorded in multiple experiments from a single cell. These experiments may need to be analyzed with different pipelines. PatchView leverages the labels (those are usually predefined by experimenters for each protocols) associated with each experiment to automatically sort data into its corresponding category. Sorted data can be directly submitted to downstream pipeline, such as mini-event analysis.

In addition to that, other software mentioned above does not support analysis for data recorded from multiple channels simultaneously. PatchView supports multi-channel analysis, such as synaptic connection analysis (Figure 3).
